# Atypical Presentation of Spontaneous Pneumomediastinum Secondary to Cannabinoid Hyperemesis Syndrome

**DOI:** 10.7759/cureus.95097

**Published:** 2025-10-21

**Authors:** Dhruv K Patel, William C Lippert

**Affiliations:** 1 Internal Medicine, Wake Forest School of Medicine, Winston-Salem, USA

**Keywords:** cannabinoid hyperemesis syndrome (chs), esophageal perforation exclusion, pulmonary laceration, spontaneous pneumomediastinum (spm), vomiting-induced barotrauma

## Abstract

Spontaneous pneumomediastinum (SPM) is a rare condition that typically affects young adult males and is often precipitated by acute increases in intrathoracic pressure.

We report a 20-year-old male with cannabinoid hyperemesis syndrome (CHS) who developed SPM. He presented with persistent vomiting, notably without classic symptoms such as chest pain, dyspnea, or subcutaneous emphysema. Imaging revealed moderate pneumomediastinum, bilateral pulmonary lacerations, and a small left apical pneumothorax. Conservative management with supportive care resulted in full clinical recovery.

This case highlights an atypical presentation of SPM and underscores the role of repetitive vomiting and recreational drug use as precipitating factors. This case also demonstrates that significant radiographic findings can occur even in minimally symptomatic patients. Awareness of atypical presentations and associated risk factors is critical for timely diagnosis and appropriate management. Clinicians should maintain a high index of suspicion for SPM in patients with CHS, even in the absence of chest pain.

## Introduction

Pneumomediastinum, defined as the presence of free air within the mediastinal structures, is a relatively common finding in hospitalized patients but can present diagnostic challenges in the emergency setting. It may occur spontaneously or secondary to trauma, iatrogenic procedures, or underlying pathology such as esophageal or airway perforation [1,2]. Spontaneous pneumomediastinum (SPM) is most often seen in young male adults and is typically precipitated by events that increase intrathoracic pressure [3]. Prompt recognition of SPM is essential due to its potential to mimic other conditions.

Cannabinoid hyperemesis syndrome (CHS) is characterized by recurrent episodes of intractable nausea, vomiting, and abdominal pain in the setting of chronic cannabis use [4]. Although its exact prevalence remains unclear, CHS has become increasingly recognized with rising cannabis use, particularly among young adults [4]. The cyclical hyperemesis phase often involves repeated vomiting and retching, which can markedly elevate intrathoracic pressure and predispose to alveolar rupture [4]. This pathophysiologic mechanism provides a plausible link between CHS and SPM.

This report presents an unusual presentation of SPM in a 20-year-old male with suspected CHS, who lacked the classic symptoms of chest pain, dyspnea, or subcutaneous emphysema. Notably, imaging revealed bilateral pulmonary lacerations and a small apical pneumothorax. Although apical pneumothorax can arise from a range of causes, including barotrauma and underlying lung disease, its occurrence alongside pulmonary lacerations in the absence of trauma is unusual. By detailing this atypical presentation, we aim to expand clinical awareness of SPM. This case also aims to contribute to the growing body of literature on SPM and reinforces the need for a high clinical suspicion in patients with nonspecific symptoms but relevant risk profiles.

## Case presentation

A 20-year-old male with no known past medical history presented to the emergency department with three days of worsening nausea and vomiting. He had no associated chest pain, dyspnea, cough, fever, or recent trauma; however, he did note a few occurrences of mild hematemesis. There was no history of prior gastrointestinal or pulmonary disease. He reported daily marijuana use, primarily via smoking, but did not disclose duration or frequency of use.

On arrival, he was hemodynamically stable with a normal blood pressure (133/66), heart rate (63 BPM), respiratory rate (18 breaths per minute), SpO2 (99% on room air), and temperature (98.2°F oral). Patient weighed 67.7 kg (149 lb 4 oz) and was 177.8 cm (5’ 10”) tall. Laboratory studies were significant for an increased total bilirubin of 1.8 (normal range: 0.3-1.0 mg/dL) and slightly decreased lipase (normal range: 11-82 U/L). All other blood work, including a comprehensive metabolic panel and a complete blood count, was unremarkable. Urine drug screening was positive for tetrahydrocannabinol (THC).

**Figure 1:**
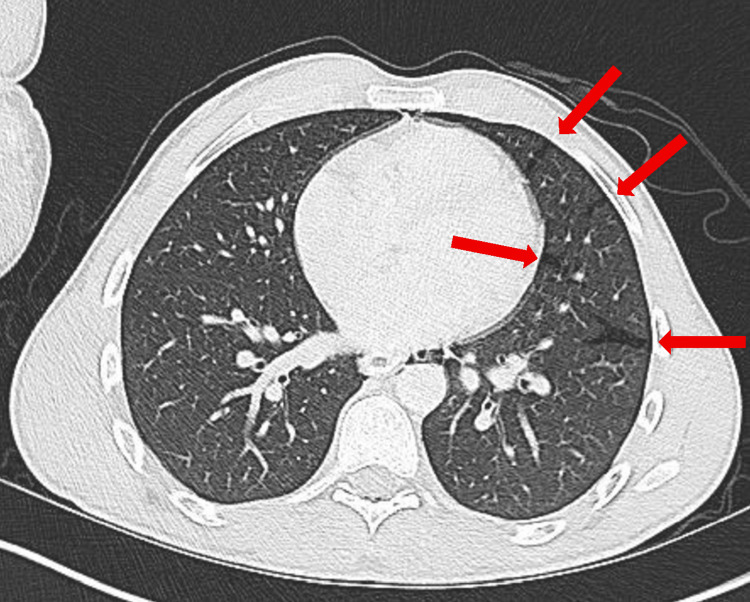
Computed Tomography (CT) scan of the chest (axial view) demonstrating pulmonary lacerations (red arrows).

**Figure 2:**
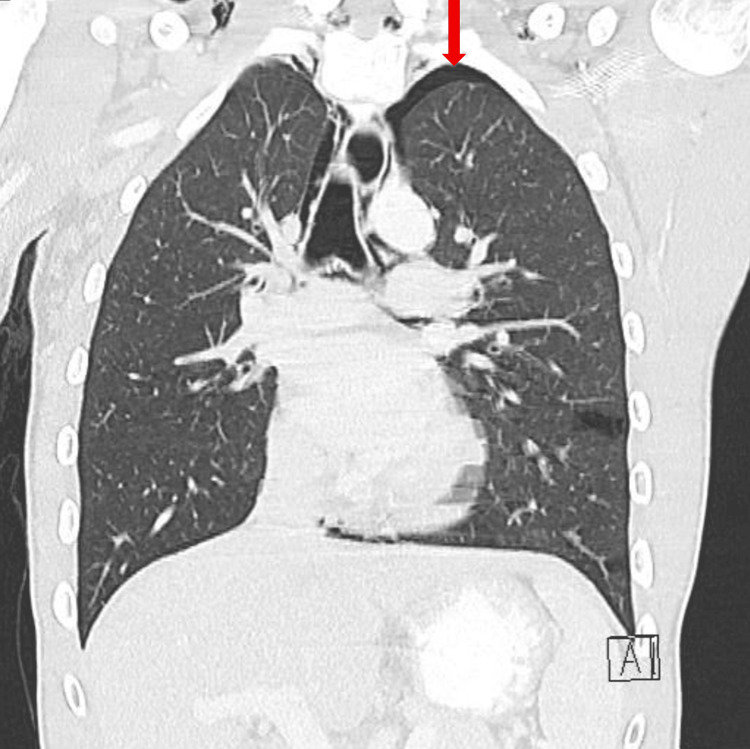
Computed Tomography (CT) scan of the chest (coronal view) demonstrating small left apical pneumothorax (less than 20%) (red arrow).

**Figure 3:**
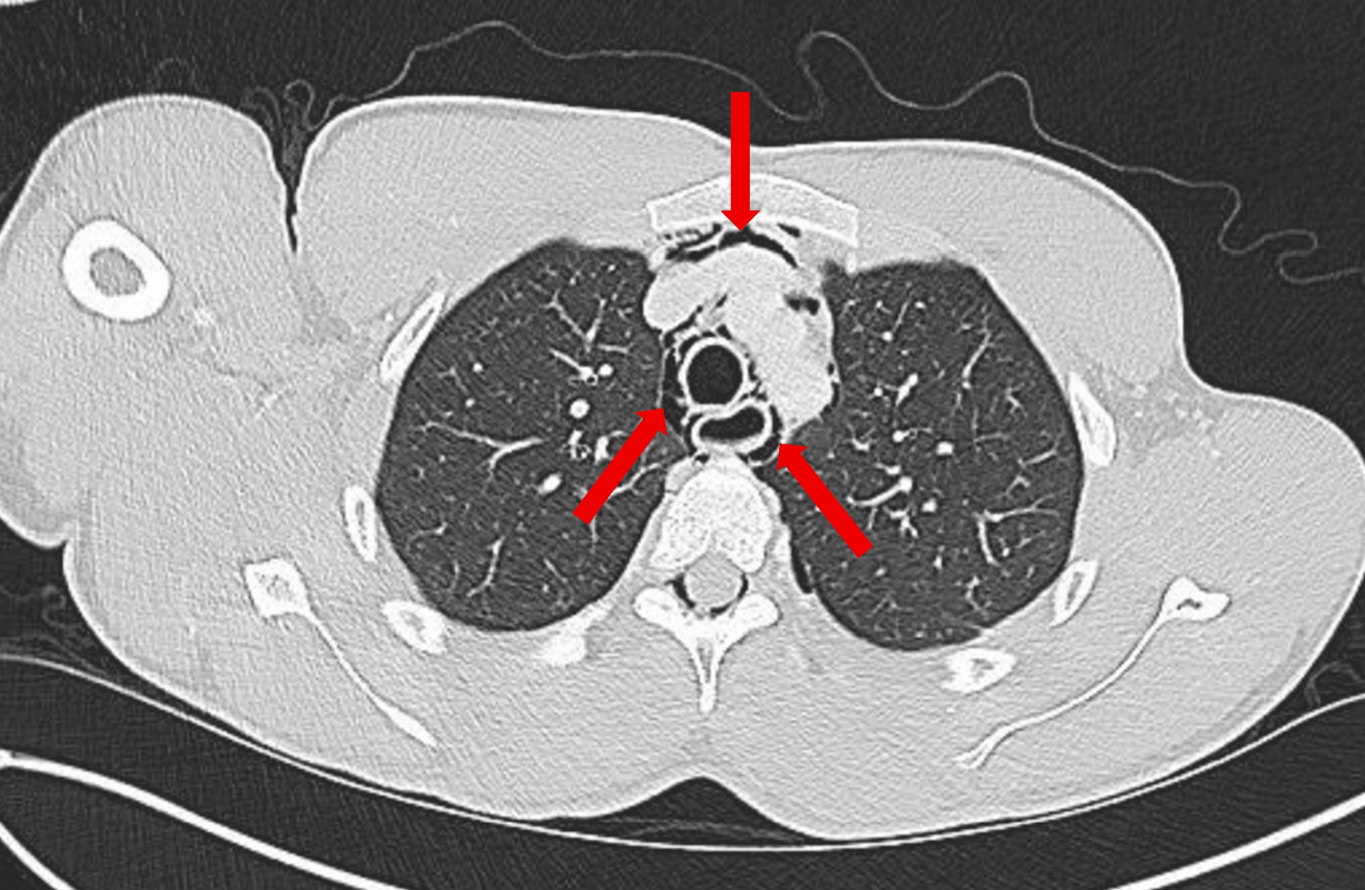
Computed Tomography (CT) scan of the chest (axial view) demonstrating moderate pneumomediastinum (red arrows).

A computed tomography (CT) scan of the abdomen and pelvis (A/P) was obtained to evaluate his gastrointestinal symptoms and incidentally revealed a partially visualized pneumomediastinum surrounding the esophagus. A subsequent CT scan of the chest demonstrated bilateral pulmonary lacerations (Figure 1), a small left apical pneumothorax (less than 20%) (Figure 2), and moderate pneumomediastinum (Figure 3), soft tissue air, and pneumorrhachis. Given concern for esophageal perforation, an esophagram was performed, which showed no evidence of esophageal perforation.

Cardiothoracic surgery was consulted and recommended conservative management. The patient was treated with IV fluids, antiemetics (ondansetron), analgesia (acetaminophen), and bowel rest with diet advancement as tolerated. He remained clinically stable throughout his hospital course. A chest X-ray obtained on the day of discharge showed persistent pneumomediastinum with clear lungs, no pleural effusion, and no pneumothorax, indicating radiographic stabilization. The patient improved without surgical intervention and was discharged home on hospital day 2 with a plan to follow up with his primary care physician in 1-2 weeks.

In the absence of an identifiable gastrointestinal etiology for his vomiting, his symptoms were attributed to cannabinoid hyperemesis syndrome, with spontaneous pneumomediastinum thought to have resulted from increased intrathoracic pressure and subsequent alveolar barotrauma.

## Discussion

Spontaneous pneumomediastinum (SPM) is characterized by the presence of free air within the mediastinum in the absence of an apparent precipitating event. It is distinct from secondary pneumomediastinum, which occurs due to trauma, iatrogenic procedures including endoscopic or dental interventions, or underlying pathology such as esophageal or airway perforation [1,2]. The incidence of SPM is approximately 1 in 30,000 emergency department referrals, based on a retrospective analysis performed at two major teaching hospitals over a five-year period [5]. SPM most commonly affects young adults, with a mean age between 17 and 25 years, and the vast majority of cases occur in individuals under 40 [3]. There is a strong male predominance among affected patients; however, the underlying gender disparity is not fully understood [3].

The pathophysiology of SPM is most commonly explained by the Macklin effect, first described in 1937 [6]. According to this mechanism, a sudden increase in intrathoracic pressure leads to alveolar rupture, allowing air to dissect along the bronchovascular sheaths into the mediastinum [6]. Murayama et al. and Morales Eslava et al. further characterize this process radiographically using CT scans, which visualize linear air collections tracking along the peribronchial and perivascular interstitium, dissecting toward the pulmonary hilum with eventual spread into the mediastinum [6,7]. Activities that acutely increase intrathoracic pressure, such as retching, vomiting, coughing, or Valsalva maneuvers, can precipitate this sequence [3,6].

In this case, the patient’s persistent vomiting, likely associated with CHS, may have generated sufficient intrathoracic pressure to cause alveolar rupture and barotrauma, leading to the pulmonary lacerations and apical pneumothorax observed on imaging. Although few cases in the literature directly link CHS to SPM, two reports by Hernández Garcia et al. and Hernández-Ramos et al. describe similar presentations in young patients with chronic cannabis use who developed SPM following episodes of severe, repetitive vomiting [8,9]. While this association remains uncommon, our case adds to the limited body of evidence supporting a potential connection between CHS and SPM. Understanding this mechanism is essential, as it provides insight into atypical presentations of SPM and helps guide appropriate diagnostic evaluation and management.

The most common presenting symptoms of SPM are consistent across studies, with both Caceres et al. and Morgan et al. reporting chest pain, dyspnea, and subcutaneous emphysema as the top three complaints [1,3]. The retrospective chart reviews also overlap regarding common precipitating events, which include retching, emesis, asthma exacerbations, coughing, tobacco use, and physical activity, with emesis and asthma flare-ups being the most frequently reported triggers [1,3]. Another, less common trigger is illicit or recreational drug use, particularly inhaled substances such as cannabis, cocaine, heroin, and tobacco [3,10]. This association is thought to result from Valsalva-type maneuvers commonly performed during drug inhalation, which acutely increase intrathoracic pressure and can precipitate alveolar rupture [10]. In the series by Macia et al., 24.3% of patients with SPM had a history of illicit drug use, including cannabis and cocaine [11]. Despite these associations, presentations of SPM can be highly variable, and some patients may be asymptomatic or present with nonspecific complaints, underscoring the importance of maintaining a high index of suspicion in at-risk populations.

While rare, vomiting-induced pneumomediastinum has been reported in the literature, including a case series of six patients initially referred for suspected esophageal perforation who were ultimately diagnosed with SPM [12]. However, all six patients experienced chest pain as a primary complaint [12]. What makes the present case significant is that although the patient presented with persistent emesis, he exhibited not only an absence of chest pain but also the three hallmark presenting symptoms of SPM. Furthermore, the presence of bilateral pulmonary lacerations is exceedingly rare in spontaneous cases, as such findings are typically associated with traumatic secondary pneumomediastinum rather than SPM [13]. These radiographic findings likely reflect the severity of barotrauma induced by repetitive vomiting in the setting of CHS.

When evaluating patients presenting with SPM, alternative causes should be carefully considered and systematically excluded. In our case, Boerhaave’s syndrome was ruled out due to the absence of severe chest pain or imaging evidence of esophageal perforation, with negative esophagography further confirming this. Traumatic causes were excluded, given the lack of recent trauma or iatrogenic procedures. An asthma exacerbation was also unlikely, as the patient had no history of asthma, wheezing, or respiratory distress, and pulmonary examination was unremarkable. While CHS was considered the most likely precipitating factor, clinicians should maintain a high index of suspicion for more common causes of pneumomediastinum in all cases.

As the case presentation demonstrates, clinical findings of SPM can often be nonspecific or absent. Therefore, the diagnostic evaluation of SPM relies heavily on imaging. Chest radiography is the standard initial modality; however, if the diagnosis remains uncertain or there is clinical suspicion for associated complications such as pneumothorax, pulmonary lacerations, or pleural effusions, CT scanning is indicated, as it is more sensitive for detecting smaller air collections and associated abnormalities [10]. Atypical features of SPM, such as fever, pleural effusion, or age over 40, should prompt further evaluation for esophageal perforation using esophagography [3]. Once the diagnosis is confirmed, management of SPM is generally conservative. Literature suggests there is no significant difference in clinical course with conservative management, regardless of the severity of SPM [14]. Supportive treatment typically includes supplemental oxygen, analgesia, antiemetics, and rest [14]. Additional interventions may be warranted on a case-by-case basis depending on clinical suspicion, comorbidities, or complications. In this patient, conservative management was pursued with favorable outcomes, further supporting the efficacy of conservative interventions even in cases with atypical clinical and radiographic findings.

Though limited, similar cases of SPM secondary to CHS, such as Hernández Garcia et al. and Hernández-Ramos et al., have been reported in the literature [8,9]. Both of these patients presented with typical symptoms, including chest pain or dyspnea, and neither had associated pulmonary lacerations or pneumothorax [8,9]. In contrast, our case is distinguished by the presence of bilateral pulmonary lacerations and a small apical pneumothorax, despite the patient being largely asymptomatic.

This case highlights several key clinical considerations about SPM. SPM can present atypically, with the absence of classic symptoms of chest pain, dyspnea, and subcutaneous emphysema, particularly in the context of CHS. This case demonstrates that significant radiographic findings, including bilateral pulmonary lacerations, can occur even in patients with minimal or no symptoms. Finally, we summarize the importance of imaging for diagnosis and reinforce that conservative management remains the mainstay of treatment, even in unusual presentations. Awareness of these features can aid clinicians in recognizing and appropriately managing cases of SPM.

## Conclusions

SPM can present atypically, particularly in patients with CHS, where significant barotrauma may occur even without classic symptoms such as chest pain or dyspnea. Early imaging is essential for timely diagnosis and exclusion of secondary causes, especially in patients with persistent vomiting. Conservative management remains the mainstay of treatment and is associated with excellent outcomes. Clinicians should counsel patients on cannabis cessation to prevent recurrence and remain vigilant for atypical presentations to ensure prompt recognition and appropriate care.
